# Effects of 4-week xylitol tablet intake on tongue microbiota composition in children: a single-arm pilot study

**DOI:** 10.1128/spectrum.01360-25

**Published:** 2025-12-23

**Authors:** Mikari Asakawa, Michiko Furuta, Shinya Asada, Tomonori Ando, Tatsuo Yanagisawa, Eiji Yoshikawa, Shinya Kageyama, Toru Takeshita

**Affiliations:** 1 Section of Preventive and Public Health Dentistry, Division of Oral Health, Growth and Development, Faculty of Dental Science, Kyushu University 12923, Fukuoka, Japan; 2 Research and Development Center, Lotte Co., Ltd., Saitama, Japan; 3 Yanagisawa Dental Office, Tokyo, Japan; 4 KSO, Co. Ltd., Tokyo, Japan; Johns Hopkins University Bloomberg School of Public Health, Baltimore, Maryland, USA

**Keywords:** xylitol, tongue microbiota, 16S rRNA, children

## Abstract

Xylitol helps reduce the levels of mutans streptococci in the oral cavity and the acidogenicity of dental plaque. This single-arm interventional pilot study investigated the effect of 4-week xylitol tablet intake on tongue microbiota composition in children. Tongue coating samples were collected during four visits, at 4-week intervals, from 23 orally healthy children aged 4–5 years, who consumed three xylitol tablets per day during the intervention period, and their bacterial composition was determined using 16S rRNA gene amplicon sequencing. The tongue microbiota shift during the intervention period was compared with that during the pre-intervention period without intake. A significantly greater microbiota shift, as measured by the Aitchison distance, was observed during the intervention period than during the pre-intervention period (*P* < 0.05, Wilcoxon signed-rank test). Following the intervention period, we detected significant reductions in the relative abundances of *Prevotella jejuni* and *Leptotrichia* HMT-417, whereas a significant increase in the relative abundance of *Granulicatella adiacens* was noted. No significant difference was observed in their relative abundances before and after the follow-up period, suggesting that the effect of xylitol tablet intake persisted for four weeks after quitting the intake. These results suggest a possibility that continuous intake of xylitol tablets helps alter the tongue microbiota composition.

**IMPORTANCE:**

Xylitol reportedly has beneficial effects on the maintenance of oral health, including the prevention of dental caries. This study focused on the effects of a 4-week xylitol tablet intake on tongue microbiota composition in children aged 4–5 years. Our results demonstrated greater changes in the bacterial composition during four weeks with xylitol tablet intake compared to those without the intake, characterized by relative abundance increases of oral health-associated taxa, such as *Granulicatella adiacens*. The results of this study suggest that continuous intake of xylitol tablets may contribute to the alteration of tongue microbiota composition to a healthy pattern.

**CLINICAL TRIALS:**

This study is registered with the UMIN Clinical Trials Registry (UMIN-CTR) as UMIN000051982.

## INTRODUCTION

The tongue dorsum occupies a large area of the intraoral surface and is colonized by a dense microbial community constituted by a diverse array of indigenous members. Tongue microbiota is mostly composed of common commensal taxa, such as *Streptococcus* and *Neisseria* species, but their relative abundances differ among individuals (1–3). Dental caries, which is a major cause of tooth loss, is undoubtedly caused by dysbiosis in the dental plaque microbiota that accumulates on the teeth (4–7). On the other hand, recent studies have reported that dental caries experience is also accompanied by a shift in the tongue microbiota characterized by high abundances of *Veillonella* and *Prevotella* species in primary school children and elderly adults (8, 9). Such interindividual variations of the tongue microbiota are also reported to be associated with systemic conditions such as an increased risk of pneumonia in the elderly (10). Given that the tongue microbiota is the primary source of the oral bacterial population ingested with saliva (11), improvement in the dysbiotic tongue microbiota has the potential to help improve and maintain oral and systemic health.

Xylitol is a naturally occurring five-carbon polyol sweetener (sugar alcohol) used in several products, including chewing gum and tablets, and its intake has been shown to be effective in improving oral health, especially in preventing dental caries. Habitual xylitol consumption decreases the levels of cariogenic bacteria, including mutans streptococci and lactobacilli, as well as the amount of dental plaque (12–16). Several studies have also reported that xylitol intake reduces the acidogenicity of dental plaque (17–19), indicating that xylitol intake also affects the environmental conditions of the intraoral cavity. However, it remains unclear whether xylitol intake alters the overall bacterial composition of tongue microbiota.

This single-arm pilot study observed a shift in the tongue microbiota of orally healthy children aged 4–5 years with a 4-week xylitol tablet intake. We determined the bacterial composition using bacterial 16S rRNA gene sequencing and compared the microbiota shift with that occurring during the pre-intervention period to identify the effect of xylitol tablet intake. This study also examined whether the shifted microbiota was transient or maintained for four weeks after discontinuing tablet intake.

## MATERIALS AND METHODS

### Study population

**Fig 1:**
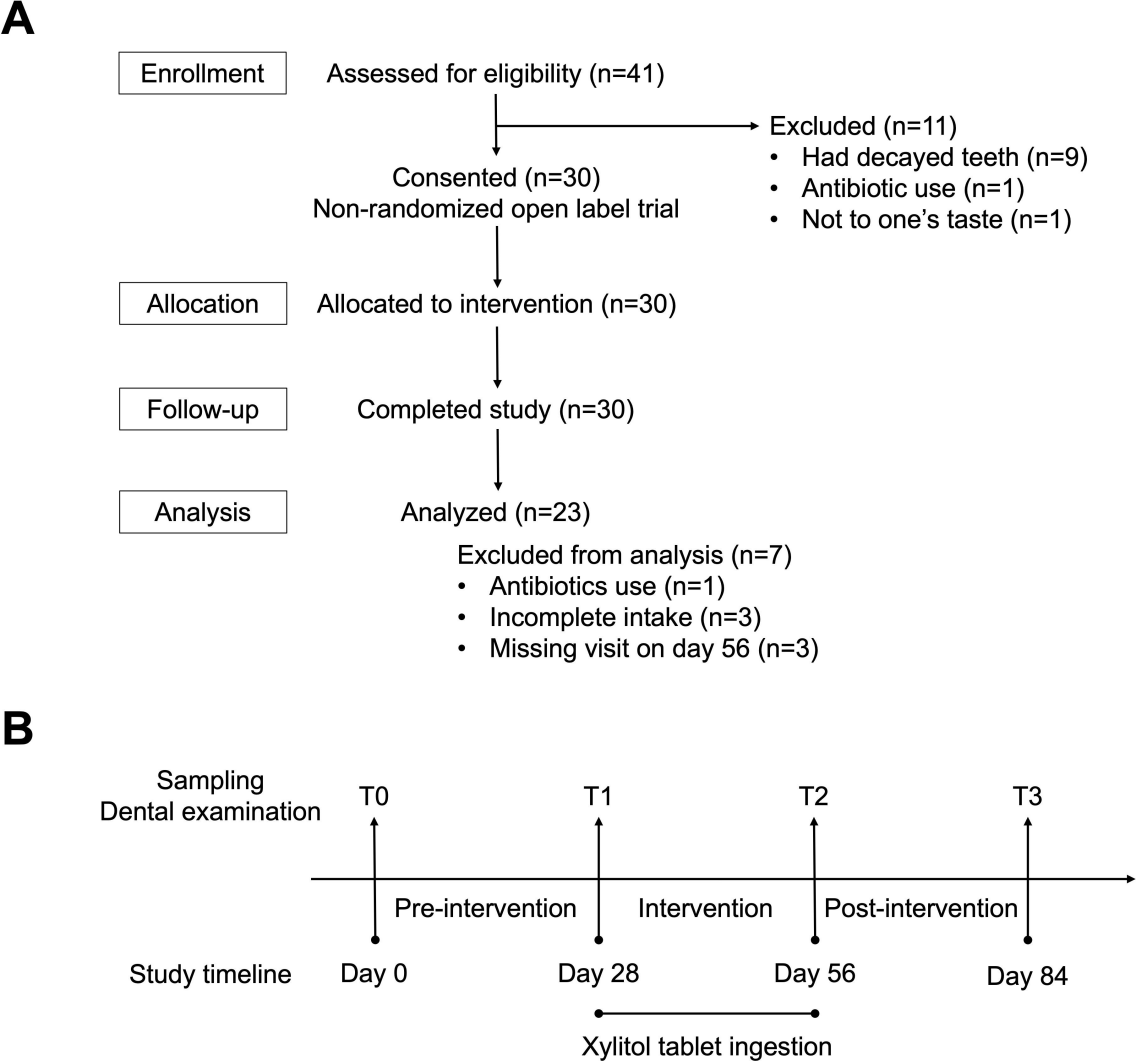
Flowchart of sample collection. (**A**) Study population selection. (**B**) Timeline of this study.

Study participants were recruited from a volunteer database associated with a contract research organization. All the participants were Japanese and lived in the Tokyo metropolitan area. Forty-one children, aged 4–5 years, participated in this single-arm interventional pilot study between September and November 2023. No prior sample size calculations were performed, and the sample size was determined based on the practical constraints of the study, rather than on statistical considerations. The criteria for inclusion were children who (i) were generally in good health (ii), had no specific diseases, and (iii) were not taking any medication. The exclusion criteria were participants with decayed teeth or receiving dental treatment, those who had difficulty licking tablets without chewing, those who disliked the taste of the tablets, and those who had used antibiotics during the 3-month period. Written informed consent was obtained from parents of all participants. The Ethics Committees of the Nihonbashi Cardiovascular Medicine Clinic in Tokyo, Japan, and of Kyushu University approved this study and the procedure for obtaining informed consent (approval numbers: NJI-023-05-01 and 20232007, respectively). The study protocol was registered in the UMIN Clinical Trials Registry (UMIN-CTR) (ID: UMIN000051982). Full details of the flow of participants in this study are shown in Fig. 1.

### Study design and intervention

In this single-arm study, with a total duration of 84 days, tongue microbiota samples were collected from each participant at four visits (T0 [day 0], T1 [day 28], T2 [day 56], and T3 [day 84]) accompanied by dental examination (Fig. 1). This study consisted of three periods: a pre-intervention period of four weeks (T0 to T1), an intervention period of four weeks (T1 to T2), and a post-intervention period of four weeks (T2 to T3). During the intervention period, the enrolled participants were instructed to ingest a xylitol tablet (1.0 g each; Lotte Co., Ltd., Tokyo, Japan) three times per day. The participants were instructed to ingest the tablet by slow licking it with the tongue and without chewing, to maximize the retention time in the oral cavity. At the first visit (T0), we checked whether the children could lick the xylitol tablet without chewing, and those who could not were excluded from the study at this point. During the intervention period, the parents of the participants were instructed to let the child lick the xylitol tablet for each intake and to record the participant’s daily intake of these tablets and other medications. We compared the tongue microbiota shifts during the intervention period with the temporal shift observed during the pre-intervention period without intake. Xylitol tablets were not ingested during the post-intervention period, and the microbiota shift was traced after quitting the intake.

### Dental examination and tongue microbiota collection

Dental examinations and the collection of tongue microbiota samples were conducted at the Yanagisawa Dental Office in Tokyo, Japan. Three trained dentists examined the number of present teeth and decayed, missing, and filled tooth surfaces (dmfs) (20) at each visit. Oral hygiene status was assessed based on the dental plaque score using a modified version of the simplified debris index, which represents the debris component of the simplified oral hygiene index (21). Dental plaque scores were examined on selected facial or lingual surfaces of the index teeth (teeth 51, 55, 65, 71, 75, and 85) (22). Following the dental examination, tongue microbiota samples were collected by swabbing the center of the tongue dorsum with a HydraFlock swab (Puritan Medical Products, Guilford, ME, USA). The collected tongue coating sample was immersed in 200 μL of lysis buffer containing 10 mM Tris-HCl, 1 mM EDTA, and 1% sodium dodecyl sulfate and was transported to the laboratory in Kyushu University on dry ice.

### 16S rRNA gene sequencing analysis

16S rRNA gene sequencing analysis was conducted at Kyushu University. DNA extraction from the tongue coating samples was performed using a bead-beating method with 0.3 g of zirconia-silica beads (0.1 mm diameter [BioSpec Products, Bartlesville, OK, USA]) and a tungsten-carbide bead (3 mm diameter [Qiagen, Hilden, Germany]), as described previously (23). 16S rRNA gene sequencing was performed using tongue coating samples collected from the participants. The V1–V2 region of the 16S rRNA gene from each sample was amplified using the following primers: 8F (5′-AGA GTT TGA TYM TGG CTC AG-3′), with Ion Torrent adaptor A and the sample-specific 8-base tag sequence, and 338R (5′-TGC TGC CTC CCG TAG GAG T-3′), with the Ion Torrent trP1 adaptor sequence. PCR amplification, purification, and quantification of each adaptor were performed as previously described (24). Emulsion PCR and enrichment of template-positive particles were performed using Ion 520 and Ion 530 Kit-OT2 (Thermo Fisher Scientific, Waltham, MA, USA) with the Ion One Touch 2 system (Thermo Fisher Scientific). The enriched particles were loaded onto an Ion 530 Chip (Thermo Fisher Scientific), and sequencing was performed on the Ion GeneStudio S5 System (Thermo Fisher Scientific) using an Ion 520 and Ion 530 Kit-OT2 (Thermo Fisher Scientific).

### Data analysis and taxonomy assignment

Raw sequence reads were quality-filtered using a script written in R (version 4.2.1). The reads were excluded from the analysis when they exhibited ≤200 bases or did not include the correct forward and reverse primer sequences. The remaining reads were demultiplexed by examining the 8-base tag sequence, and the forward and reverse primer sequences were trimmed. Quality-checked reads were further processed using the dada2 package (version 1.32.0) in R, including quality filtering, denoising, and chimera filtering procedures with settings for Ion Torrent reads, and an amplicon sequence variant (ASV) table was produced (25). Seventeen ASVs observed in the negative control and corresponding to *Pseudomonas fluorescens* were excluded from the ASV table and subsequent analyses as PCR contaminants. The taxonomy of each sequence was initially determined using the RDP classifier, with a minimum support threshold of 80%, and RDP taxonomic nomenclature (to the genus level) (26). Subsequently, species-level taxonomic assignment was performed using BLAST (27) against 1,015 oral bacterial 16S rRNA gene sequences (16S rRNA RefSeq version 15.22) in the expanded Human Oral Microbiome Database (eHOMD) (28). Nearest-neighbor species with ≥98.5% identity were selected as candidates for each sequence. In addition, we constructed a species-level table from the ASV table by clustering the ASVs assigned to the same reference sequence in eHOMD. The relative abundance of each taxon was calculated from a non-rarefied feature table at the genus and species levels. The Shannon index and phylogenetic diversity (PD) were calculated following rarefaction at a depth of 5,000 reads per sample using R.

### Statistical analysis

All statistical analyses were performed using R software (ver. 4.2.1). The Wilcoxon signed-rank test was used to compare the alpha diversity indices during each period or visit.

Differentially abundant taxa at each visit were identified using MaAsLin2 (Microbiome Multivariable Associations with Linear Models) (29), based on the CLR-transformed genus- and species-level tables. When using MaAsLin2, features with a low relative abundance (<1%) were trimmed prior to model fitting. Comparisons of the relative abundance shifts of predominant taxa with a mean relative abundance of ≥1% were performed using the difference in features after CLR transformation, and significance was calculated using the Wilcoxon signed-rank test. *P* values were adjusted using the Benjamini–Hochberg method for multiple comparisons. The Friedman test was used to assess the differences in dental plaque scores at each visit. The dissimilarity in bacterial composition among the samples was evaluated using the Aitchison distance based on centered log-ratio-transformed abundance data at the species level (30). The distances between each visit-point pair were compared using the Wilcoxon signed-rank test. Permutational multivariate analysis of variance (PERMANOVA) based on the Aitchison distance was used to assess the relationship between each visit point and tongue microbiota composition, using the Adonis function in the vegan library.

## RESULTS

### Characteristics of participants

We screened 41 children for eligibility and subsequently excluded 11. Of the 30 participants enrolled in this study, seven were excluded from the analysis due to antibiotic use during the intervention period, incomplete intake of xylitol tablets (the intake was missed on one or more days), and missing visits on day 56 (Fig. 1). Of the 23 participants analyzed, one underwent a dental examination and tongue microbiota collection on day 90 instead of day 84. The general and clinical characteristics of the participants are shown in Table 1. During the study period, the development of dental caries was not observed in any participant. Eruption of permanent teeth occurred in one participant. No significant difference was observed among dental plaque scores of the participants at four visits (0.4 ± 0.4, 0.3 ± 0.4, 0.4 ± 0.4, and 0.4 ± 0.4; *P* = 0.576).

**TABLE 1:** Characteristics of individuals

Characteristics	Mean ± SD or frequency
Age (mean ± SD)	4.6 ± 0.5
Woman (*n*, %)	10 (43.5)
Weaning age (*n*, %)	
<12 months	4 (17.4)
12–17 months	12 (52.2)
≥18 months	7 (30.4)
Siblings (*n*, %)	19 (82.6)
Smoking status of father (*n*, %)	
Never	11 (47.8)
Past	10 (43.5)
Current	2 (8.7)
Smoking status of mother (*n*, %)	
Never	22 (95.7)
Past	1 (4.4)
Current	0 (0)
Toothbrushing frequency (*n*, %)	
One time/day	7 (30.4)
Two times/day	12 (52.2)
Three times/day	4 (17.4)
Toothpaste use (*n*, %)	
Never	4 (17.4)
Without fluoride	1 (4.4)
With fluoride	18 (78.2)
Brushing of teeth by mother (*n*, %)	23 (100.0)
Regular dental check-ups^*a*^ (*n*, %)	17 (73.9)

^*a*^ Regularly visit a dentist for oral care at least once a year.

### An effect of 4-week xylitol tablets intake on the tongue microbiota

Bacterial composition of 92 tongue microbiota samples (23 participants and four visits) was determined by 16S rRNA gene sequencing, which provided 1,757,866 denoised reads (18,537 ± 7,739 reads per sample), comprising 2,316 ASVs. Of all ASVs, 1,460 ASVs (91.2% of all reads) exhibited ≥98.5% identity with the reference sequences in eHOMD and were clustered into 234 taxa at the species level.

**Fig 2:**
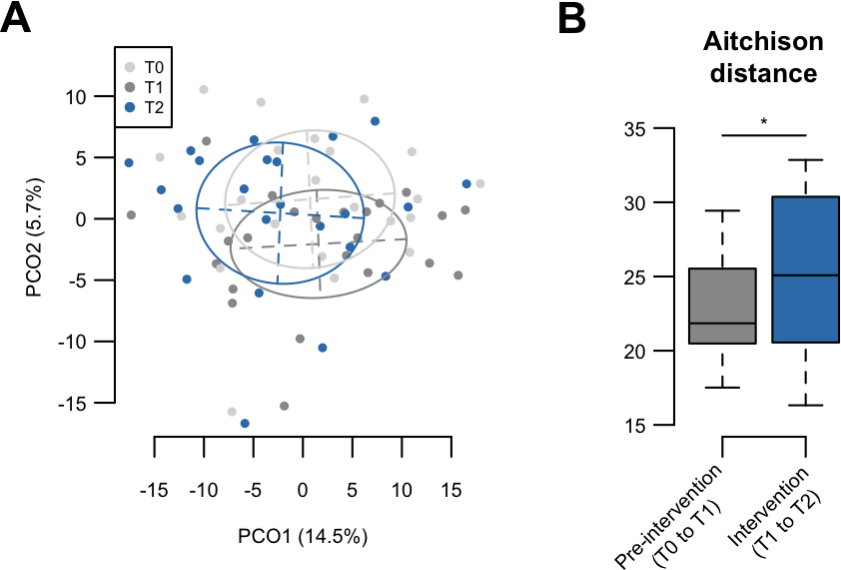
Compositional changes of tongue microbiota during pre-intervention and intervention periods. (**A**) Principal coordinate analysis plot of each time point using the Aitchison distance at the species level. Bacterial compositions of tongue coating samples at T0, T1, and T2 are depicted in different colors. Ellipses cover 67% of samples belonging to each time point. (**B**) Aitchison distance of pre-intervention (T0 to T1) and intervention (T1 to T2) periods. Significance was calculated using the Wilcoxon signed-rank test. *P < 0.05.

**Fig 3:**
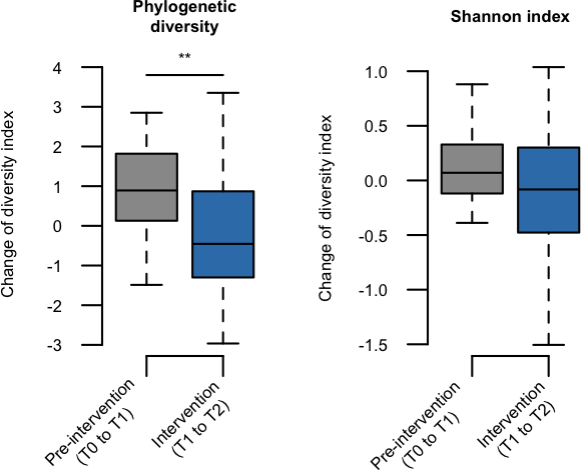
Changes in alpha diversity indices of tongue microbiota during pre-intervention and intervention periods. Changes in phylogenetic diversity and Shannon indices during pre-intervention (values of T1 minus T0) and intervention (values of T2 minus T1) periods are shown. Significance was calculated using the Wilcoxon signed-rank test. **P < 0.01.

The tongue microbiota of children aged 4–5 years is not assumed to have matured completely, considering a previous result (31); therefore, the microbiota of these participants is also expected to change according to the maturation process. Thus, this single-arm study compared the microbiota shift in the intervention period (T1 to T2) with that in the pre-intervention period (T0 to T1) without xylitol tablet intake. A principal coordinate analysis (PCoA) plot based on the Aitchison distance index showed a gradual shift in the bacterial composition from T0 to T2 (Fig. 2A), whereas the distances in the intervention period were significantly greater than those in the pre-intervention period (Fig. 2B). It has been suggested that a 4-week xylitol tablet intake has a substantial effect on tongue microbiota composition. Of the two microbial diversity indices, the increase in phylogenetic diversity was significantly lower during the intervention period (T1 to T2) than during the pre-intervention period (T0 to T1) (Fig. 3).

**Fig 4:**
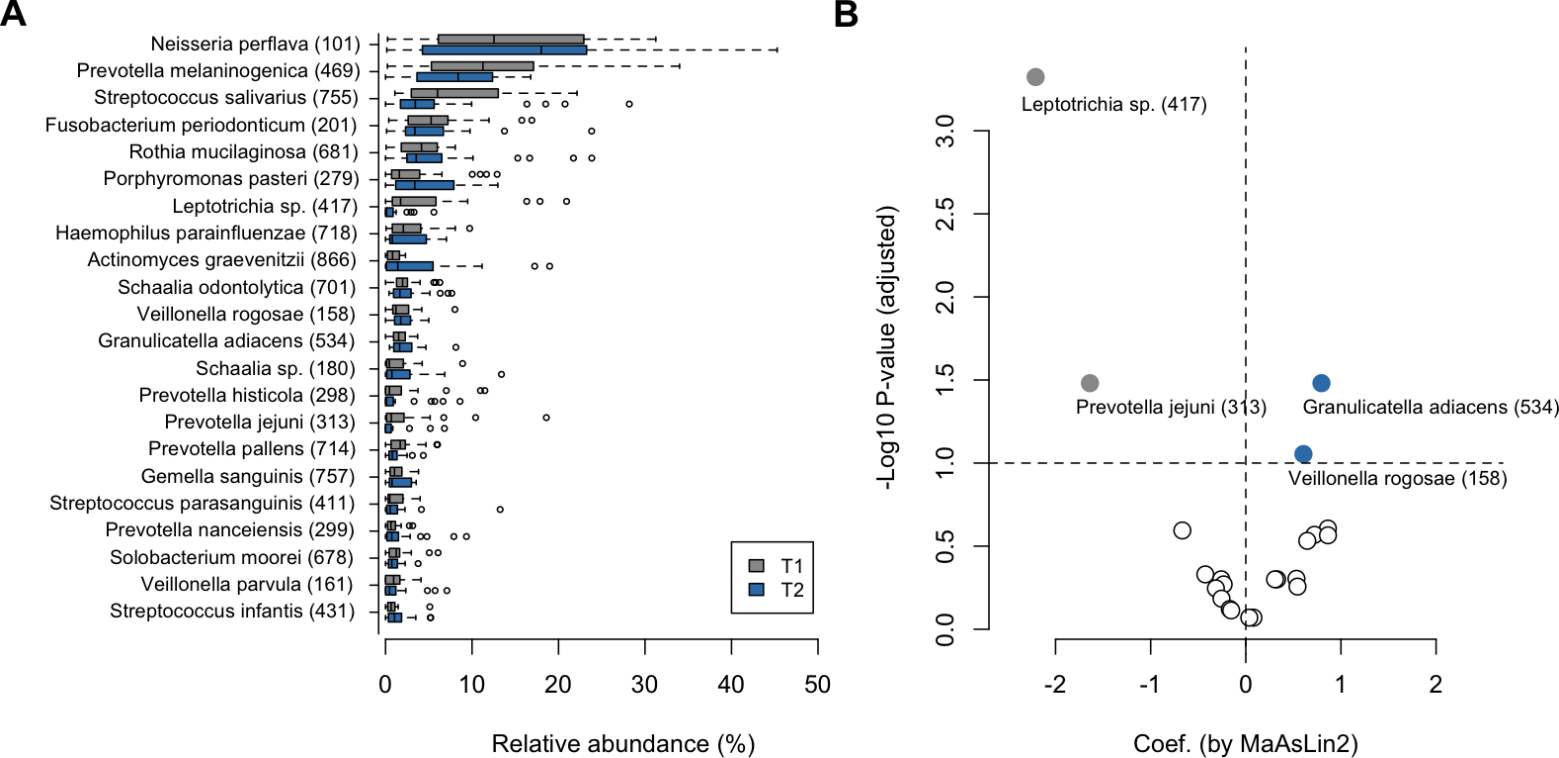
Differences in the tongue microbiota at the species level before and after intervention. (**A**) Relative abundance of 22 predominant species among the tongue microbiota before and after intervention. (**B**) Species differentially abundant before and after intervention based on MaAsLin2. Dots indicate the 22 predominant species, the *x*-axis shows the effect size (coefficient), and the *y*-axis represents –log10 (adjusted *P* value). Threshold: adjusted *P* value < 0.1. Negative values indicate species that were more abundant prior to intervention (gray), and positive values indicate species more abundant after intervention (blue).

To identify the bacterial taxa that characteristically changed in abundance from the pre- to post-intervention periods, we compared the microbiota composition at T1 and T2 using MaAsLin2, a discriminant analysis tool for compositional data based on the CLR-transformed genus- and species-level tables. Of the 16 genera with a mean relative abundance of ≥1%, *Leptotrichia* became less predominant in the microbiota after the intervention period, whereas *Granulicatella* became more predominant (Fig. S1). At the species level, we detected a significant decline in the relative abundances of *Leptotrichia* HMT-417 and *Prevotella jejuni* after the intervention period (Fig. 4A and B). In contrast, the relative abundances of *Granulicatella adiacens* and *Veillonella rogosae* significantly increased following the intervention period. We confirmed that these changes during the intervention period (T1 to T2) were significantly greater than those observed during the pre-intervention period (T0 to T1) (Fig. S2).

### Persistence of effect of xylitol tablet intake

**Fig 5:**
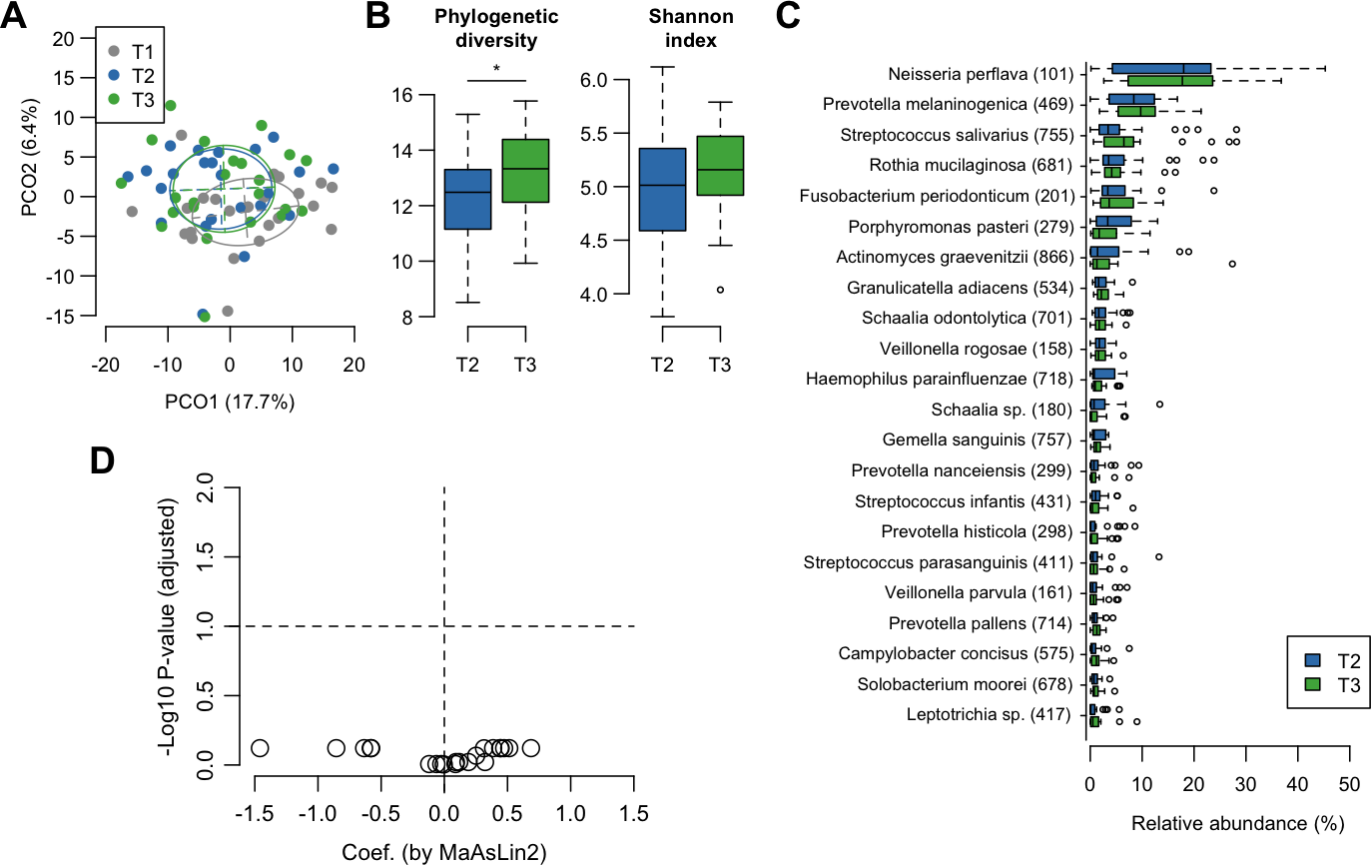
Persistence of the effect of xylitol tablet intake. (**A**) Principal coordinate analysis plot of each time point using Aitchison distance at the species level. Bacterial compositions of tongue coating samples at T1, T2, and T3 are depicted in different colors. Ellipses cover 67% of samples belonging to each time point. (**B**) Alpha diversity indices of tongue microbiota before and after follow-up period. Significance was calculated using the Wilcoxon signed-rank test. *P < 0.05. (**C**) Relative abundance of predominant species in tongue microbiota before and after follow-up period. Taxon IDs in the eHOMD database are given in parentheses following bacterial names. (**D**) Species differentially abundant before and after the follow-up period based on MaAsLin2. Dots indicate the 22 predominant species. The *x*-axis shows the effect size (coefficient), and the *y*-axis represents –log10 (adjusted *P* value). Threshold: adjusted *P* value < 0.1.

The microbiota shift was further traced for four weeks after cessation of xylitol tablet intake. A PCoA plot based on Aitchison distance showed that the points corresponding to the tongue microbiota at T3 remained different from those at T1 (Fig. 5A). Phylogenetic diversity indices that decreased during the intervention period significantly increased after the follow-up period (Fig. 5B). However, no significant differences were observed in the relative abundances of the predominant taxa, including *Prevotella*, *Leptotrichia*, *Granulicatella*, and *Veillonella* species between T2 and T3 (Fig. 5C and D). A comparison among each visit point revealed that the relative abundance of *Leptotrichia* HMT-417 was significantly lower after intervention (T2) than before (T1) (Fig. 4B), and this trend was found to be maintained when assessed at the end of the follow-up period (T3) (Fig. S3). These results suggest that shifted microbiota during the intervention period were generally maintained even after a 4-week follow-up period.

## DISCUSSION

This single-arm study demonstrated compositional shifts in the tongue microbiota of 4- and 5-year-old children during the 4-week intervention period with xylitol tablet intake, characterized by a decrease in the relative abundance of *Leptotrichia* HMT-417 and *P. jejuni*, and an increase in *G. adiacens* and *V. rogosae* (Fig. 4B). Our previous studies on 138 primary school children and 506 community-dwelling elderly adults revealed that the tongue microbiota is composed of two cohabiting groups of predominant commensals and that a high ratio of commensal taxa, including *Prevotella* and *Leptotrichia* species, is associated with more teeth affected by dental caries and poorer dental hygiene (8, 9). A recent study from another laboratory also showed that the salivary and dental plaque microbiota dominated by *Prevotella* and *Leptotrichia* species were observed prior to the clinical detection of carious lesions (32). A decrease in the relative abundance of these taxa would be implicated in a lower risk of dental caries. In contrast, *G. adiacens,* which increased in this study, was included among another group of commensal taxa in the above-mentioned studies (8, 9) that had higher relative abundances in individuals with healthy dental conditions (8, 9, 24). *Granulicatella* species have also been reported to be predominant in the oral microbiota of healthy individuals compared to that in patients with type 2 diabetes or reflux esophagitis (33, 34). Although it is unlikely that the subtle shift in tongue microbiota composition observed in this study improves actual oral conditions, these results suggest a possibility that continuous xylitol tablet intake helps alter the tongue microbiota composition to a healthy pattern.

This study further observed a shift in the tongue microbiota for four weeks after cessation of xylitol tablet intake, but no significant differences were observed between the relative abundances of the 22 predominant taxa, including the abovementioned five taxa, before and after the follow-up period (Fig. 5C and D). A PCoA plot based on the Aitchison distance also showed that the tongue microbiota after the 4-week follow-up period differed from that of the pre-intervention period (Fig. 5A). A previous double-blind randomized controlled trial showed that 15 days of xylitol administration reduced *Streptococcus mutans* levels, and the reduction persisted for a period of three months after discontinuation (35). Another study indicated that 13 months of xylitol chewing gum use reduced levels of salivary mutans streptococci, and this trend was still observed after a 3-month cessation (36). Our data strengthened the notion that the effects of continuous xylitol intake persisted in the tongue microbiota for a certain period after cessation.

Phylogenetic diversity, which is an index of microbial richness that takes into account the phylogenetic information of each species, significantly decreased during the intervention period (Fig. 3). It is also observed in an Estonian study, which showed that xylitol intake reduced the number of observed bacterial phylotypes in the salivary microbiome (37). The intake of xylitol tablets may alter the environmental conditions in the intraoral cavity, with high selection pressure for microorganisms. Interestingly, phylogenetic diversity recovered during the follow-up period (Fig. 5B) suggests that such an effect persists only during exposure. Future studies focusing on the gene expression and metabolites that occur in the tongue microbiota in the presence of xylitol may be helpful in identifying the components associated with the survival of minority taxa in the oral microbiota.

In this study, well-known caries-related bacteria, such as *Streptococcus mutans* and *Streptococcus sobrinus*, were not detected in any of the assessed participants. In addition, *Lactobacillus* was detected in only a single participant and at a very low relative abundance (≤0.14%). We speculate that these findings may be attributable to the fact that the specimens used in this study were tongue coating samples rather than dental plaque, and that individuals with decayed teeth or those receiving dental treatment were not included in the analysis.

The tongue microbiota comprises a microbial community that is distinct from that of dental plaque (2). Thus, it remains to be established how xylitol influences its bacterial composition. The potential mechanisms underlying xylitol-mediated regulation of the tongue microbiota are assumed to be associated with an inhibition of bacterial metabolism. For example, xylitol has been shown to inhibit bacterial glycolysis and suppress the growth of bacteria such as mutans streptococci (38, 39). In addition, the findings of previous studies have revealed that xylitol prevents bacterial adhesion to surfaces, thereby inhibiting biofilm formation (40, 41), and this effect may have contributed to reductions in the abundance of mid- and late colonizers such as *Prevotella* and *Leptotrichia* (Fig. 4B) (42–44). In this regard, omics analyses, such as metagenomics and metabolomics, are needed to elucidate the detailed mechanisms whereby xylitol regulates the tongue microbiota. While numerous studies have indicated that xylitol inhibits the growth and biofilm formation of cariogenic bacteria, such as *S. mutans*, only a few studies have evaluated the effects of xylitol on oral commensal bacteria. Our findings in this study provide additional evidence to indicate that xylitol might influence the composition of oral commensal microbiota.

Despite its strengths, this study does have several limitations. Firstly, it was a single-arm study that did not include a control group, and, consequently, this study design limited causal attribution of the direct effects of xylitol. We hypothesized that the temporal shift in the tongue microbiota occurring in the 4-week intervention period would be equal to that in the 4-week pre-intervention period. However, seasonal and other changes in dietary habitats and lifestyles between the two periods could have affected the shifts in the tongue microbiota composition. Although tooth eruption was observed in only a single participant throughout the study period, such events associated with oral development might also influence shifts in microbial composition. Secondly, the sample size of this pilot study was small (23 participants), and thus subtle changes in the microbiota that were less pronounced than the corresponding inter-individual variations may have been missed. A randomized controlled trial with a larger sample size is required to verify and further clarify the details of the microbiota shift following xylitol tablet intake. Thirdly, although dental examinations were conducted by three trained dentists who could accurately evaluate oral conditions, inter-rater agreement, as determined by indices such as Cohen’s kappa and ICC, was not assessed in this study. Fourthly, although parents were instructed to let the participant lick the xylitol tablets and record their daily intake, it cannot be conclusively guaranteed that the tablets were invariably ingested without chewing. Finally, this study was designed as a short-term intervention for xylitol intake. Therefore, future studies on prolonged xylitol exposure are needed to determine its long-term effects on the tongue microbiota.

The present study demonstrated that greater changes in bacterial composition occurred during xylitol tablet consumption, particularly in health-related taxa such as *G. adiacens,* and that these changes persisted for a certain period after quitting xylitol intake. These findings suggest a possibility that continuous xylitol intake helps maintain oral and systemic health by affecting the tongue microbiota composition.

## Data Availability

Sequence data were deposited in the DDBJ Sequence Read Archive under the accession number PRJDB35964.

## SUPPLEMENTAL MATERIAL

Supplemental material spectrum.01360-25-s0001.docx Figures S1 to S3.

ASM does not own the copyrights to Supplemental Material that may be linked to, or accessed through, an article. The authors have granted ASM a non-exclusive, world-wide license to publish the Supplemental Material files. Please contact the corresponding author directly for reuse.
