# Supplementary material for: Effects of 4-week xylitol tablet intake on tongue microbiota composition in children: a single-arm pilot study
Source: Microbiol Spectr. 2025 Dec 23;14(2):e01360-25. doi: 10.1128/spectrum.01360-25 (PMC12889142; doi:10.1128/spectrum.01360-25)
Supplement: Supplemental material — Figures S1 to S3. [file spectrum.01360-25-s0001.docx]

Supplementary Appendix

Effects of 4-week xylitol tablet intake on tongue microbiota composition in children: a single-arm pilot study

Mikari Asakawa^a^, Michiko Furuta^a^, Shinya Asada^b^, Tomonori Ando^b^, Tatsuo Yanagisawa^c^, Eiji Yoshikawa^d^, Shinya Kageyama^a^, Toru Takeshita^a^*

^a^Section of Preventive and Public Health Dentistry, Division of Oral Health, Growth and Development, Faculty of Dental Science, Kyushu University, Fukuoka, Japan

^b^Research and Development Center, Lotte Co., Ltd., Saitama, Japan

^c^Yanagisawa Dental Office, Tokyo, Japan

^d^KSO, Co. Ltd., Tokyo, Japan

*Corresponding author: Toru Takeshita, taketooo@dent.kyushu-u.ac.jp

Contents:

Fig. S1. Differences in the tongue microbiota at the genus level before and after intervention.

Fig. S2. Changes in relative abundance of predominant species in tongue microbiota during pre-intervention and intervention periods.

Fig. S3. Relative abundances of predominant taxa in tongue microbiota at each visit.

**Fig. S1. Differences in the tongue microbiota at the genus level before and after intervention.**


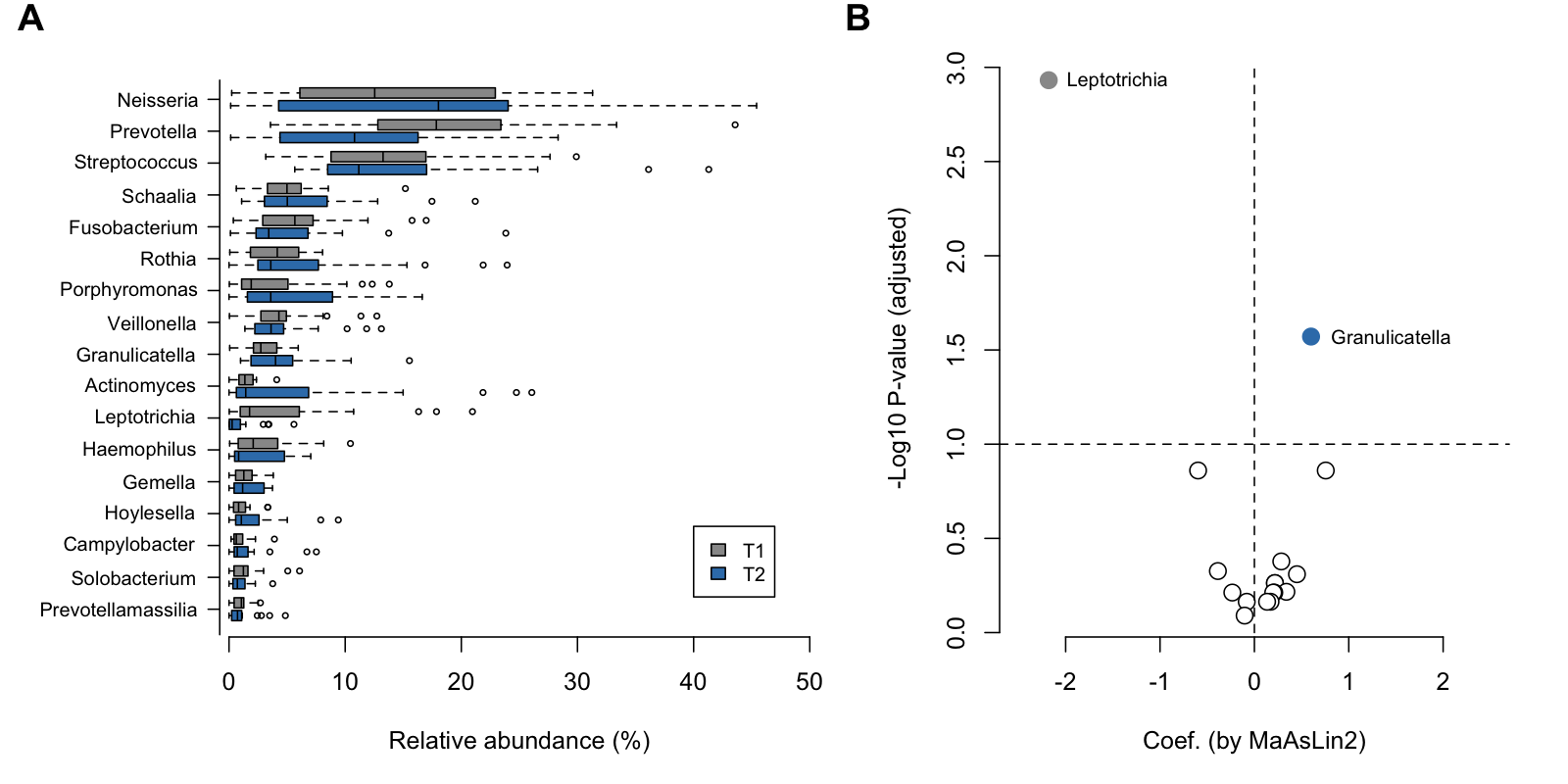


(A) Relative abundance of 17 predominant genera among the tongue microbiota before and after intervention. (B) Genera differentially abundant before and after intervention based on MaAsLin2. Dots indicate the 17 predominant genera, the x-axis shows the effect size (coefficient), and the y-axis represents -log10 (adjusted *P* value). Threshold: adjusted *P* value < 0.1. Negative values indicate genera that were more abundant prior to intervention (gray) and positive values indicate genera more abundant after intervention (blue).

**Fig. S2. Changes in relative abundance of predominant species in tongue microbiota during pre-intervention and intervention periods.**


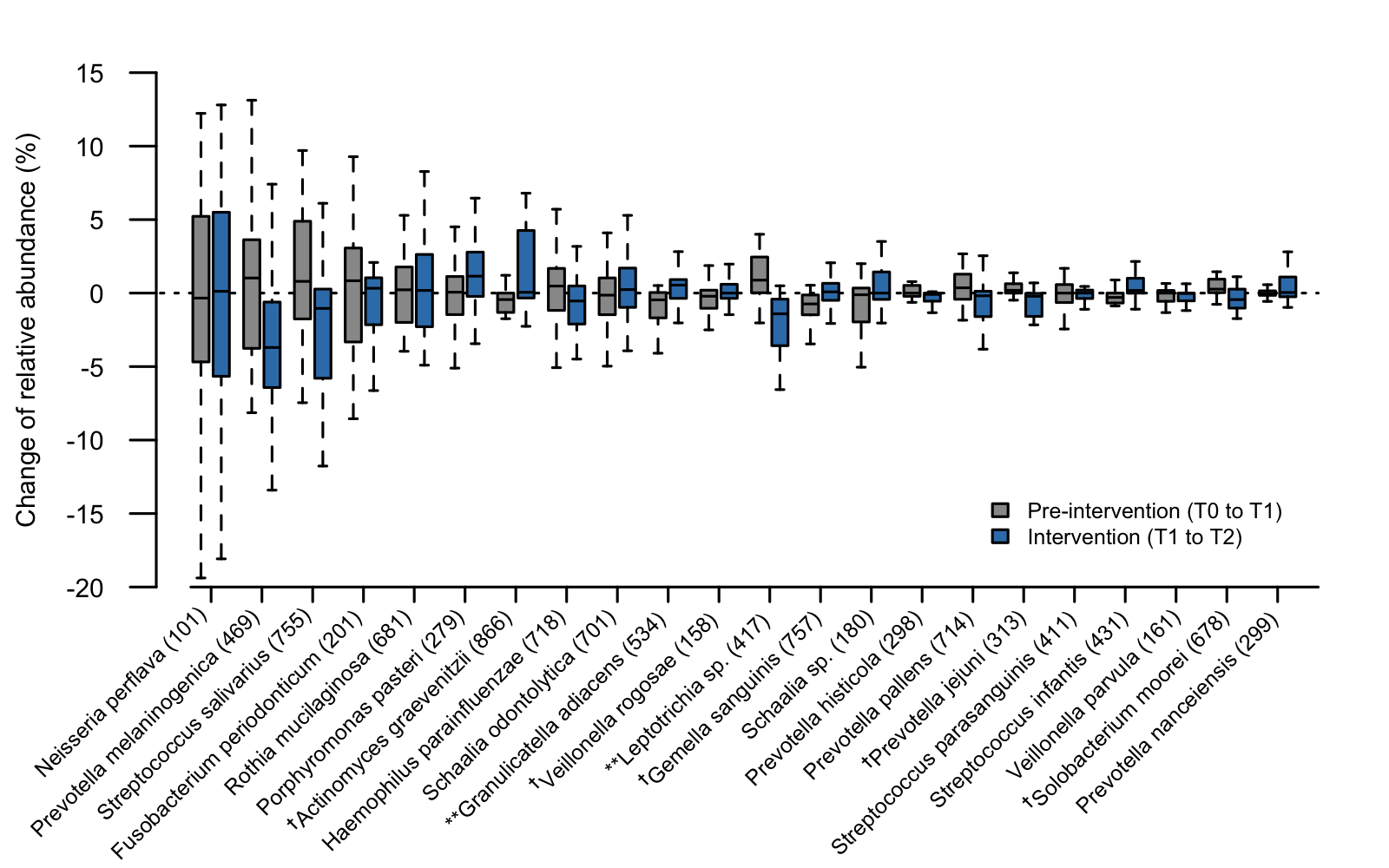


Changes in relative abundance of 22 species with a mean relative abundance ≥ 1% during pre-intervention (values of T1 minus T0) and intervention (values of T2 minus T1) periods are shown. The differences in the CLR-transformed abundances were compared and significance was calculated using Wilcoxon signed-rank test. ^†^*P* < 0.1, ^*^*P* < 0.05, ^**^*P* < 0.001 after Benjamini-Hochberg adjustment.

**Fig. S3. Relative abundances of predominant taxa in tongue microbiota at each visit.**


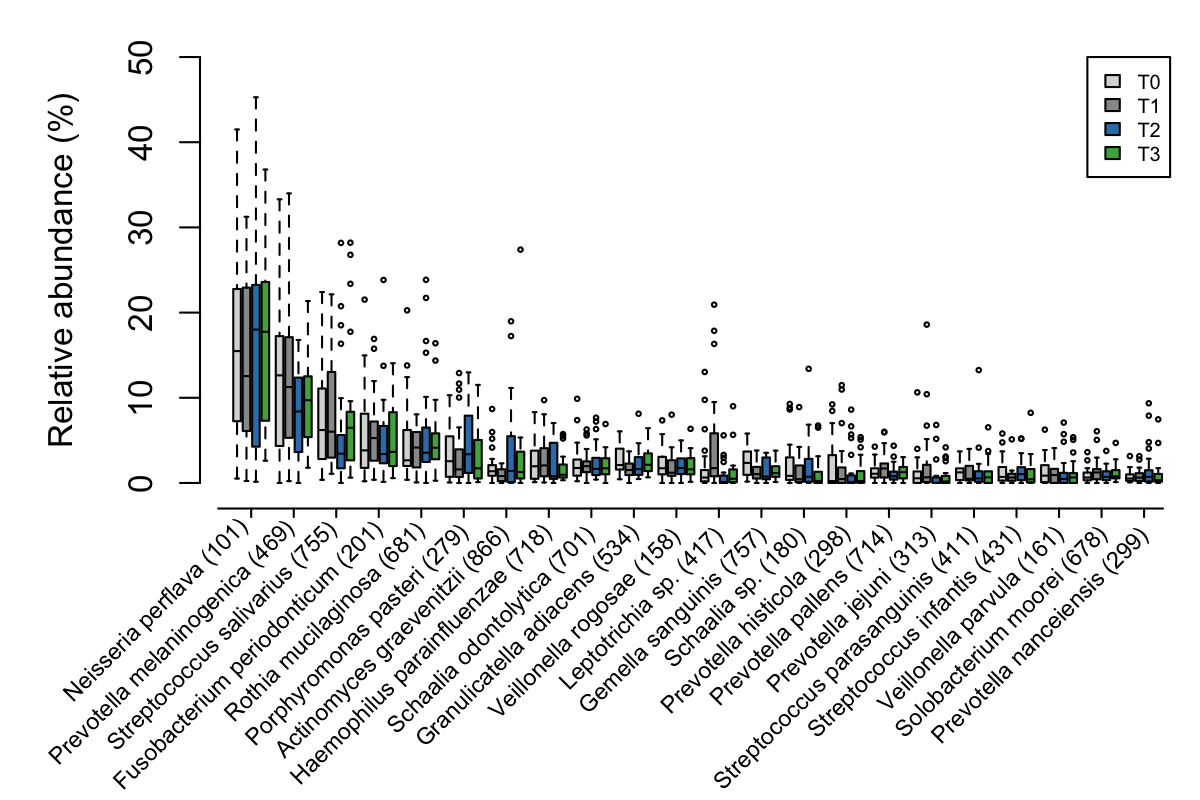


Relative abundance of 22 predominant with a mean relative abundance ≥ 1% at each visit are shown.
